# Accounting for contact network uncertainty in epidemic inferences with Approximate Bayesian Computation

**DOI:** 10.1007/s41109-025-00694-y

**Published:** 2025-04-22

**Authors:** Maxwell H. Wang, Jukka-Pekka Onnela

**Affiliations:** Department of Biostatistics, Harvard University, 677 Huntington Ave, Boston, MA 02115 USA

**Keywords:** Networks, Approximate Bayesian Computation, SIR model

## Abstract

In models of infectious disease dynamics, the incorporation of contact network information allows for the capture of the non-randomness and heterogeneity of realistic contact patterns. Oftentimes, it is assumed that this underlying network is known with perfect certainty. However, in realistic settings, the observed data usually serves as an imperfect proxy of the actual contact patterns in the population. Furthermore, event times in observed epidemics are not perfectly recorded; individual infection and recovery times are often missing. In order to conduct accurate inferences on parameters of contagion spread, it is crucial to incorporate these sources of uncertainty. In this paper, we propose the use of Network-augmented Mixture Density Network-compressed ABC (NA-MDN-ABC) to learn informative summary statistics for the available data. This method will allow for Bayesian inference on the parameters of a contagious process, while accounting for imperfect observations on the epidemic and the contact network. We will demonstrate the use of this method on simulated epidemics and networks, and extend this framework to analyze the spread of Tattoo Skin Disease (TSD) among bottlenose dolphins in Shark Bay, Australia.

## Introduction

In the study of emerging epidemics, it is important to understand the individual-level transmission dynamics of the contagion. Individual-based models can capture high resolution features of disease transmission patterns, and can be useful for evaluating interventions that act on the individual level or predicting the spread of new contagions. To capture the dynamics of an epidemic, models attempt to describe the spread of disease via a set of mechanistic rules, which are typically modulated via a set of parameters that abstract the various aspects of disease transmission. For example, the per-contact transmissibility may capture the instantaneous rate of transmission across paths of potential transmission (Newman 2002; Wilson et al. 2008; Groendyke et al. 2012), and the expected duration of infectiousness captures the average time an individual remains infectious before recovery. Other parameters may capture the increase or decrease of viral load in an individual over time (Vrijens et al. 2005; Jarvis and Kelley 2021), the decrease in the probability of infection associated with protective mask-wearing (Bai and Brauer 2021), or the proportion of individuals that are asymptomatic carriers of the contagion (Aguilar et al. 2020; Luo et al. 2021). Thus, an accurate disease model requires estimates of a range of parameters, as well as the quantification of the uncertainty associated with the estimates. Such estimation must often proceed from observed data, which is often subject to various sources of noise and missingness that are inherent to real-world data collection.

For this purpose, it is natural to consider estimation and inference from a Bayesian perspective, which focuses on the distribution of parameters conditional on the observed data and allows for the incorporation of prior information. In this paper, we focus on inferences that utilize data exhibiting two sources of uncertainty commonly present in observational studies: uncertainty regarding the contact structure of the population under study, and uncertainty regarding the contagion event times (e.g., times of infection and recovery).

The contact structure of a population is naturally captured by a contact network, which represents individuals as nodes and potential transmission paths as edges. Such networks have been used to model HIV (Kretzschmar and Wiessing 1998; Sloot et al. 2008; Vieira et al. 2010), measles (Groendyke et al. 2012), COVID-19 (Liu et al. 2020; Hambridge et al. 2021; Tetteh et al. 2021), and non-biological contagions, and contact networks are useful for identifying high-value targets for targeted interventions (Pastor-Satorras and Vespignani 2002; Cohen et al. 2003), informing methods to modify contact patterns to prevent contagion spread (Bu et al. 2013; Youssef and Scoglio 2013; Zhang et al. 2022), or as a predictor for the development of contagion (Harling et al. 2017; Wang et al. 2023). However, there remains a need for methods that provide a statistically informed way to incorporate the uncertainty and missingness present in real-world network observations in disease models. Oftentimes, observed contact data serves as an informative proxy for the underlying patterns of contact, but the true contact network is not known with full certainty. For example, social survey data may capture a reported relationship, but this perceived social link may only serve as a proxy for the true underlying contact type of interest, such as the duration of time two individuals spend within two meters of each other each week.

Secondly, the full history of the epidemic under study is rarely fully observed. In particular, the times of infection and recovery for individuals may not be known, especially if the contagion of interest is asymptomatic, or if the status of individuals cannot be ascertained at all time points. Oftentimes, the disease status of individuals in the population may be known only at discrete points in time from diagnostic testing (such as for COVID-19) or observations of the presence of symptoms.

A common method to account for missing data is data-augmented Markov Chain Monte Carlo (MCMC). In these settings, unknown variables, such as missing event times, are treated as latent parameters that are jointly inferred upon alongside the parameters of interest. Such MCMC methods have been applied to a variety of applications in network epidemics (Britton and O’Neill 2002; Groendyke et al. 2011, 2012; Embar et al. 2014; Bu et al. 2022). However, when large amounts of data are missing, the latent variable space can become very high-dimensional. In such settings, MCMC may not be computationally tractable unless the expressiveness of the model for either the epidemic or the network observations is appropriately constrained.

Approximate Bayesian Computation (ABC) is another subset of methods for Bayesian inference, first developed in the study of genetics (Tavaré et al. 1997; Beaumont et al. 2002). Under the ABC paradigm, results are forward-simulated from the model, given a proposal value of the parameters, and these results are compared to the observed data. Proposal parameter values are accepted if the simulation results they produce are deemed to be similar enough to the observed data. In order to maintain computational tractability while also avoiding poor results from the curse of dimensionality (Blum 2010), the similarity of results is usually evaluated as the difference between two sets of summary statistics. As the algorithm does not require the specification of a likelihood, ABC is especially useful when considering models that are analytically complex, yet simple to describe in terms of mechanistic rules.

In this paper, we will focus on an augmented version of the Mixture Density Network-compressed ABC (MDN-ABC) (Hoffmann and Onnela 2022). This method has been used for epidemic inferences on complex models when the contact network is fully observed (Wang and Onnela 2023), but can be expanded to scenarios where the contact network is an additional parameter that must also be inferred. Unlike many other ABC approaches that depend on summary statistics evaluated from the trajectory of the epidemic, our method circumvents the need for summary statistic selection. Instead, MDN-ABC utilizes informative summary statistics extracted from a mixture density network (Bishop 1994), a neural network which learns the component weights and parameters of a mixture density model. By augmenting the MDN-ABC procedure with an additional network sampling step introduced in Young et al. (2020), our method allows for sampling from the joint posterior distribution of the unknown network and the contagion parameters of interest. Previous methods that considered similar problems tend to focus on specific models for the contact network, such as simple statistical models (Britton and O’Neill 2002; Bu et al. 2022) or spatial models (Almutiry and Deardon 2020). Contact network-augmented MDN-ABC (NA-MDN-ABC) provides a more generalized framework that allows for a high degree of flexibility in user specification of both the model for contagion dynamics, as well as the model for observations on the contact network.

## Methods

### MDN-ABC

Approximate Bayesian Computation (ABC) is a likelihood-free method first applied to problems in population genetics (Fu and Li 1997; Tavaré et al. 1997), and has been extended to various applications in studying contagious disease (Blum and Tran 2010; Drovandi and Pettitt 2011; Neal 2012; Dutta et al. 2018; Sun et al. 2015; Almutiry and Deardon 2020).

The general idea behind ABC is to sample from the posterior distribution by accepting proposed parameter values that lead to simulated outcomes deemed similar to the observed data. Here, we define *S*(*Y*) as a summary statistic computed from the observed data *Y*, and $\delta$ as a acceptance threshold that determines how “close” a simulated dataset must be to the observed dataset for the proposed parameter value $\theta ^*$ to be accepted. A discrepancy function, $d(x,x')$, is used to calculate the $\delta$ between two datasets; here, we will use the Euclidean distance as the discrepancy function. A typical rejection ABC algorithm then follows a simple rejection sampling scheme:

**Algorithm 1:**
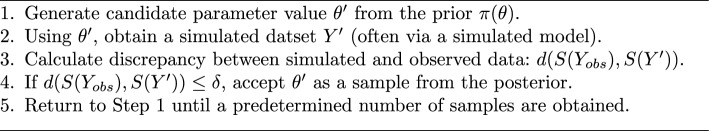
Rejection ABC

However, in many applications of ABC, the choice for the summary statistic *S*(*Y*) is not obvious. It is common to adopt *ad hoc* summary statistics that intuitively provide information on the parameters of interest, based on metrics calculated from the observed data (Dutta et al. 2018; Almutiry and Deardon 2020), or to select a best subset from a set of candidate summary statistics (Joyce and Marjoram 2008; Nunes and Balding 2010; Raynal et al. 2019). While these methods can produce readily interpretable results, it is often unclear whether or not the original pool of candidate summary statistics contains the statistics most informative for the parameters of interest.

Other methods seek to transform the observed data into lower-dimensional summary statistics while preserving critical information. These include estimation of the posterior mean as a summary statistic (Prangle et al. 2014), maximizing Fisher information (Charnock et al. 2018), or information-theoretic approaches such as minimizing Kullback–Leibler divergence (Chan et al. 2018), minimizing posterior entropy (Nunes and Balding 2010), or maximizing mutual information (Chen et al. 2020). It has been recently shown that the information-theoretic approaches are equivalent or special cases of minimizing the Expected Posterior Entropy (EPE) (Hoffmann and Onnela 2022).

In this paper, we consider the MDN-compressed ABC proposed in Hoffmann and Onnela (2022), which learns informative summary statistics by minimizing the Monte Carlo estimate of the EPE:

1 $$\begin{aligned} \hat{{\mathcal {H}}} = -m^{-1}\sum _{i=1}^m \text{ log }f(\theta _i, s(Y_i)), \end{aligned}$$

where $f(\theta ,t)$ is a conditional density estimator that approximates the posterior, $\theta _i$ and $Y_i$ are joint samples from $p(\theta ,Y)$, and *m* is the number of samples in the minibatch. This work is closely related to conditional density estimation, where a Mixture Density Network (Bishop 1994) is used to learn a conditional posterior density estimation that minimizes the EPE (Papamakarios and Murray 2016). For example, an MDN that learns a Gaussian mixture would have the component weights, as well as the means and variances of each component, as the outputs of the neural network. Conditional density estimation yields a parameterized mixture model that can approximate the posterior density arbitrarily closely, given unlimited computational power. However, conditional density estimators rely on parametric assumptions about the posterior distribution and, as such, do not enjoy the same asymptotic guarantees as ABC. Furthermore, in “NA-MDN-ABC for network inferences” section, we will augment the MDN-ABC with an additional sampling step to account for the network observation model; in order to marginalize over the high-dimensional contact networks, it is more convenient to draw samples from the posterior, rather than approximate a parameterized mixture distribution. Thus, instead of utilizing the components of the conditional density estimator, we will extract a single layer of the mixture density network to utilize as the summary statistics for ABC. This approach, dubbed the MDN-ABC, combines MDN and ABC methods to learn informative summary statistics using the MDN, while using traditional ABC sampling. Further details for this method can be found in Hoffmann and Onnela (2022) and Wang and Onnela (2023). In the following sections, we will consider augmenting the MDN-ABC to account for uncertainty in contact network reporting.

### Related work

ABC has been utilized for inferences on a wide range of epidemiological models. Compartmental models segment the population into their respective disease statuses (e.g., Susceptible, Infected, Recovered, etc.), and describe the dynamics of the epidemic as a series of differential equations that model the transitions between the various disease states (Kermack and McKendrick 1927). These models remain popular for studying transmissible disease, and ABC is readily applied for inferences on parameters within compartmental models, often using disease incidence over time as the summary statistic (Toni et al. 2009; Blum and Tran 2010; Drovandi and Pettitt 2011; Lu et al. 2013; Sun et al. 2015; Smith and Gröhn 2015; Cunha Jr et al. 2023).

One assumption of compartmental models is that the population is uniformly mixed; that is, every individual in the population has an equal probability of contacting and infecting any other individual. However, realistic contact patterns tend to exhibit non-randomness based on factors such as geographic location or social behaviors. This heterogeneity can oftentimes be modelled by further subdividing the population geographically (Brown et al. 2018; Chong et al. 2018), or as household units (Neal 2012; Kypraios et al. 2017). While these models can provide greater accuracy in describing many real-world populations, the uniform mixing assumption still holds within each individual unit (e.g., a single household).

Networks allow for even more granularity for expressing heterogeneous contact patterns, as contacts in networks can be described at an individual level. In a typical network model for an infectious disease, an infected node will only be able to transmit the infection to its network neighbors. Thus, the structure of the contact network is an important factor in determining the trajectory of an epidemic (Newman 2002; Ganesh et al. 2005; Pastor-Satorras et al. 2015). However, while recent work has utilized ABC for inferences on network epidemics (Walker et al. 2010; Dutta et al. 2018; Almutiry and Deardon 2020; Wang and Onnela 2023), most methods either assume a fully observed network or utilize relatively simple random network models. There remains a need for ABC methods that can produce inferences on parameters of a disease while also incorporating the uncertainty associated with observed network data in a statistically principled manner.

### Notation

In the remainder of the paper, we will make use of the following notations. *Y* is the observed epidemic data. We will consider cases where *Y* is a binary vector of positive and negative test results of disease status; however, it is straightforward to consider other types of outcomes, such as event times or viral loads. $\theta$ are the parameters of interest, which determine contagion dynamics. These include parameters such as the per-contact transmissibility or the mean duration of symptoms.

*A* is the true contact network that the epidemic propagates on. Note that *A* captures a network of “potential” transmission events, such that the path of transmissions is a subgraph within *A*. *X* is the observed network data, which is assumed to take the form of measurements between pairs of individuals. For example, $X_{ij}$ could represent the number of times individual *i* and individual *j* were seen to interact during the duration of the study. While *X* is informative of *A*, it does not perfectly capture *A*. Note also that while $X_{ij}$ may be defined on any suitable space (e.g., natural numbers, positive real numbers, etc.), in this paper, we assume that the network *A* is unweighted, such that $A_{ij}\in \{0,1\}$ for all pairs (*i*, *j*). Lastly, $\phi$ represents the parameters determining the distribution of the observed network data *X*, conditional on the true network *A*.

### NA-MDN-ABC for network inferences

While MDN-ABC can readily be applied to epidemics on known networks (Wang and Onnela 2023), it is often necessary to consider the uncertainty introduced by imperfect observations on networks. The unknown contact network can be considered a latent parameter that can be jointly inferred on, alongside the contagion parameters of interest. While ABC can potentially be expanded to high-dimensional applications, it is usually only practical to apply ABC when the parameter space is relatively low-dimensional. Thus, we propose focusing primarily on inferences for the contagion parameters while considering the true network as a nuisance parameter that is marginalized over. This approach is sensible if the purpose of estimation of disease parameters is to parameterize contagion models for the prediction of epidemic spread, especially if the model is meant to be generalizable over populations that exhibit differing contact structures but are affected by the same contagion.

Young et al. (Young et al. 2020) discuss a simple framework for drawing Bayesian inferences on networks when only a proxy for the true network of interest is observed; similar ideas have also been previously explored in Butts (2003) and Newman (2018). This approach consists of two primary components. First, the “data model” captures the probability distribution of the observed data, conditional on the true network: $P(X|A, \phi )$. Second, the “network model” captures the *a priori* probability of any given network: *P*(*A*). This approach is computationally straightforward when full dyadic independence can be assumed. Namely, in the data model, observations on each dyad should be independent of all other dyads. In the network model, the probability of any edge existing must be independent of any other edge existing (this includes models such as the random graph, the configuration model, and the Stochastic Block Model). Under these independence assumptions, one can express the full likelihood of $P(X|A, \phi )$ as a factorizable product of likelihoods; this leads to relatively simple sampling of the joint posterior $P(A, \phi |X)$.

In our paper, we are primarily interested in the joint posterior of the contagion parameters $\theta$, but the true network *A* and the network parameters $\phi$ must also be included to account for uncertainty in network reporting. Thus, the full posterior is in the form of $P(\theta ,A,\phi |X,Y)$, where *X* is the observed dyad-level data for the network and *Y* is the observed data for the epidemic. To extract the marginal posterior of $\theta$, one simply marginalizes over *A* and $\phi$. In the ABC setting, this marginalization is relatively simple to perform; joint samples of $(\theta , A, \phi )$ are drawn from $P(\theta ,A,\phi |X,Y)$, and we keep only the samples of $\theta$.

To proceed further, we make the following independence assumptions: (1) $X \perp Y |A$, (2) $\theta \perp \phi$, and (3) $\theta \perp A$. Here, Assumption 1 proposes that conditional on the true network, the observed network data *X* is independent of the observed epidemic data. Essentially, if the true network is known, *X* provides no additional information on *Y*, and vice versa. Note that this assumption would not hold true in situations where surveillance on the contact network is dependent on the disease status of individuals (for example, when contact tracing for a disease). Assumptions 2 and 3 imply that in the absence of any observed information, the prior distribution of $\theta$ is independent of the nature of the true network, and how that network is observed. A common scenario where these assumptions may not hold is when individuals change their contact patterns based on disease status; for example, during the COVID-19 pandemic, some individuals likely self-isolated upon experiencing symptoms. However, Assumptions 2 and 3 are reasonable when (1) individuals do not change their behavior based on disease status or (2) network inferences focus specifically on the contact network *prior* to the spread of contagion, and later behavioral changes are incorporated as dynamic evolutions of the pre-epidemic contact network. When these independence conditions are fulfilled, it is possible to consider the joint posterior in the simplified form:

2 $$\begin{aligned} P(\theta , A, \phi |X,Y) \propto P(Y|A,\theta )P(A,\phi |X)P(\theta ). \end{aligned}$$

A derivation of this formula is found in the “Appendix”. This formulation lends itself to a straightforward ABC-based sampling scheme, which is described in Algorithm 2.

**Algorithm 2:**
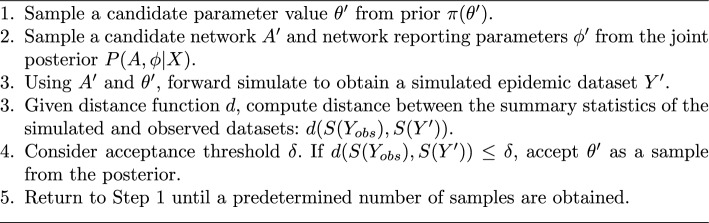
Network-Augmented Rejection ABC

**Fig. 1:**
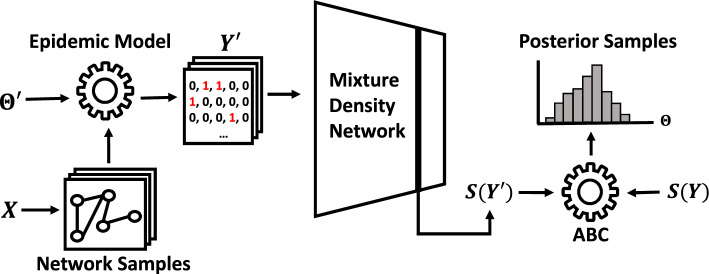
Using the framework from Young et al. (2020), network samples $A'$ are drawn based on observed network data *X*. Proposal contagion parameter values $\theta '$ are drawn from the prior. $A'$ and $\theta '$ are passed through the epidemic model to generate a simulated output $Y'$. A summary statistic of $Y'$ is calculated via an MDN, yielding $S(Y')$. Finally, $S(Y')$ is compared to the summary statistics yielded by the original observed epidemic *Y*. If the acceptance condition is fulfilled, such that $d(S(Y'),S(Y)) \le \delta$, $\theta '$ and $A'$ are accepted as part of the MDN-ABC posterior. To generate additional posterior samples, simply repeat the process with new values of $\theta '$ and $A'$

Since $P(A,\phi |X)$ is computationally tractable given some dyadic independence assumptions (Young et al. 2020), there are a variety of ways to sample proposal networks for Step 2, including simple Gibbs sampling. Following (Young et al. 2020), we implemented this model in STAN, which utilizes a Hamiltonian Monte Carlo algorithm (Carpenter et al. 2017).

In addition, in order to perform this algorithm, we must first obtain the summary statistics for *S*(*Y*). Following the MDN-ABC method defined in Hoffmann and Onnela (2022), we train an MDN by minimizing the Monte Carlo estimate of the EPE for $\theta$, the contagion parameters of interest. A single layer of this MDN is then utilized as the summary statistics for ABC. While our MDN-ABC algorithm proceeds with a simple rejection ABC algorithm, once the summary statistics are trained, application of more sophisticated ABC algorithms is straightforward.

We refer to the full procedure, which augments an MDN-ABC step with a network sampling step, as Network-Augmented MDN-ABC (NA-MDN-ABC). A diagram of the full NA-MDN-ABC method is shown in Fig. 1.

## Simulation study

In this section, we will consider a simulated SIR epidemic where the contact network and the event times are not observed.

We define the simulated population as a set of $n=100$ individuals $N = {1,\ldots ,n}$, which are represented as nodes in the network *A*. The potential paths of transmission are represented by edges $E \subseteq N \times N$. We will consider two scenarios for degree distributions of the underlying network: an Erdős–Rényi network (Erdos and Rényi 1960) with mean degree 4, and a Log-normal network with mean degree 4, which captures the heavy-tailed degree distribution often seen in realistic networks with heterogeneous contact patterns. The Erdős–Rényi network is generated as a random network, and the Log-normal network is generated using a Chung–Lu model (Chung and Lu 2002), with the parameter $\sigma ^2$ fixed at 0.5.

For our contagion, we employ a compartmental Susceptible-Infected-Recovered (SIR) model, commonly used to simulate infectious diseases. Nodes begin in a “susceptible” state, with a subset of the population initiating in the “infected” state. Infected nodes cause neighboring susceptible nodes to progress to the infected state with a per-contact transmission rate $\beta$. Infected nodes also progress to the “recovered” state with recovery rate $\gamma$. Recovered nodes no longer infect other nodes and are also unable to become re-infected themselves. More formally, if we define $W^i_t$ as the status of node *i* and time *t*, we can define $\beta$ and $\gamma$ as:

3 $$\begin{aligned} \gamma= & \lim _{\Delta t\rightarrow 0} \frac{P(W_{t+\Delta t}^i = R | W_t^i = I)}{\Delta t}. \nonumber \\ \beta= & \lim _{\Delta t\rightarrow 0} \frac{P(W_{t+\Delta t}^i = I \text{ via } j | W_t^i = S, W_t^j = I, A_{ij}=1)}{\Delta t}. \end{aligned}$$

To simulate uncertainty on epidemic event times, we will consider observations on the disease status. We periodically observe the binary disease status of each node: “infected” for nodes in the susceptible or recovered state, and “not infected” for nodes in the infected state. Thus, *Y* takes the form of a vector of binary values.

Under this formulation, one can proceed with data-augmented MCMC sampling of the posterior distribution of $\beta$ and $\gamma$ when the contact network is known (Bu et al. 2022). However, we will consider the case where the contact network *A* is not directly observed. Instead, we observe a proxy, *X*, for the contact network. For our simulated observation model, we consider *X* to be $N \times N$ matrix of dyadic encounters, such that $X_{ij}$ is the number of times nodes *i* and node *j* interact during the period of the study. We model $X_{ij}$ as a Poisson-distributed count with a rate dependent on whether or not the dyad in the true network is an edge or a non-edge, such that:

4 $$\begin{aligned} X_{ij}|A_{ij}= & 0 \sim \text{ Poisson }(\lambda _0). \nonumber \\ X_{ij}|A_{ij}= & 1 \sim \text{ Poisson }(\lambda _1). \end{aligned}$$

Furthermore, the *a priori* probability of any edge existing is considered to be a constant $\rho$:

5 $$\begin{aligned} A_{ij} \sim \text{ Bernoulli }(\rho ). \end{aligned}$$

We define $\theta = (\beta , \gamma )$ and $\phi = (\lambda _0, \lambda _1, \rho )$. The true values of the parameters were set to $\beta = 0.15$, $\gamma = 0.1$, $\lambda _0 = 1$, and $\lambda _1 = 8$. To begin each simulation, we randomly select 5 nodes as the origin of the infection. We continue our simulated epidemic for 50 timesteps, and obtain each node’s status every 7 timesteps. The status of the node is considered to be “1” if the node is infected, and “0” if the node is susceptible or recovered. If each timestep is considered to be a day, this would correspond to a weekly testing cadence. We will generate a single instance of this simulation to serve as the “true” epidemic – our observations on the true epidemic will be *Y*, the vector of binary values of individual-level test results, and *X*, the count of interactions between each pair of individuals in the population.

### MDN-ABC settings

The priors for both $\beta$ and $\gamma$ are set to $\text{ Gamma }(2,4)$ distributions. The prior for $\lambda _0$ was set to $\text{ Gamma }(2,4)$, while the prior for $\lambda _1$ is set to $\text{ Gamma }(24,2)$. The prior for $\rho$ was set to $\text{ Beta }(2,2)$. Priors were selected to place a reasonable amount of probability density at the true values of parameters, while also not being centered at the true values.

In order to train the MDN, we will generate $5\times 10^6$ samples for training, and $2.5\times 10^6$ samples for validation. As described in “Methods” section, we will draw contagion parameters $\theta$ from their priors. In order to sample contact networks for the simulations, we will utilize the HMC method described in Young et al. (2020).

For our MDN, we utilize two gamma-distributed components, which respect the supports of parameters $\beta$ and $\gamma$. Our neural network is a fully-connected architecture, with 6 hidden layers. We will extract the last hidden layer, with 15 neurons, as the vector of summary statistics. This architecture is similar to the one utilized in Wang and Onnela (2023), where it was found that a wide range of architectures for the MDN led to near-identical results. For training, we use an Adam optimizer with a learning rate of $5\times 10^{-5}$ and calculate loss on the validation set at every epoch. Once 10 epochs elapse without a decrease in validation loss, training is terminated. With the summary statistics are trained, we can re-utilize the training samples in a simple rejection-ABC algorithm. We define the discrepancy function to be the Euclidean distance.

The summary statistic vector for each training sample is compared to the summary statistics yielded by the original “true” epidemic. The 0.02% of training samples corresponding the the lowest discrepancy value are accepted as part of our MDN-ABC posterior. This corresponds to roughly 1000 posterior samples. In the next subsection, we will consider the results of the NA-MDN-ABC inference. To demonstrate the use of an alternative ABC algorithm, in the “Appendix”, we also show the posterior distributions obtained using SMC-ABC (Toni et al. 2009) instead of rejection ABC for our ABC sampling step.

### Simulation results

**Fig. 2:**
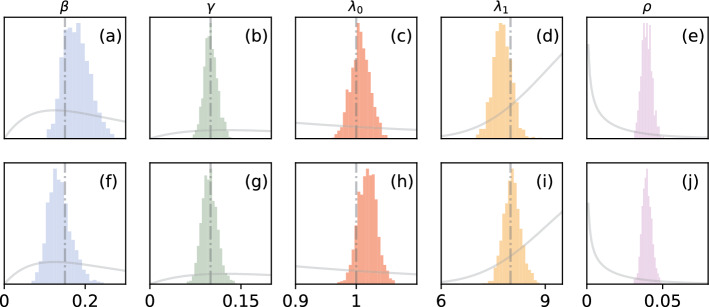
Posterior samples generated from NA-MDN-ABC. Panels **b**–**e** display the results for the Erdős–Rényi network, and panels **f**–**j** show results for the log-normal distributed network. “True” values are marked with a vertical line. Prior densities are shown in gray (prior densities for $\lambda _0$ and $\lambda _1$ have been multiplied by an additional factor of 10 for visibility)

In Fig. 2, we plot the marginal posterior densities of disease parameters $\beta$ and $\gamma$, as well as the network parameters $\lambda _0$ and $\lambda _1$, for both network scenarios (Erdős–Rényi and Log-normal degree distributions).

**Fig. 3:**
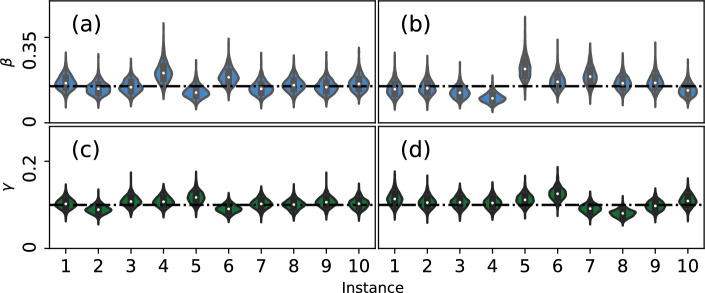
NA-MDN-ABC results across 10 instances of the original stochastic epidemic for $\beta$ and $\gamma$ on a Erdős–Rényi network (**a** and **c**) and for $\beta$ and $\gamma$ on a log-normal network (**b** and **d**). True values for parameters are marked with a horizontal line

The posterior distributions shown in Fig. 2 are much less diffuse than the prior distributions used for the parameters and tend to be roughly centered around the true values used in the simulation. However, note that the posterior distribution depends on the “original” realization of the simulated epidemic that we use as ground truth. Because this simulation is stochastic in nature, it is possible for the posterior distribution to look vastly different for varying instances of the original epidemic. To visualize this variance, we re-simulate the original epidemic 10 times for each scenario, and re-draw samples using the contact NA-MDN-ABC. We show these distributions in Fig. 3.

**Fig. 4:**
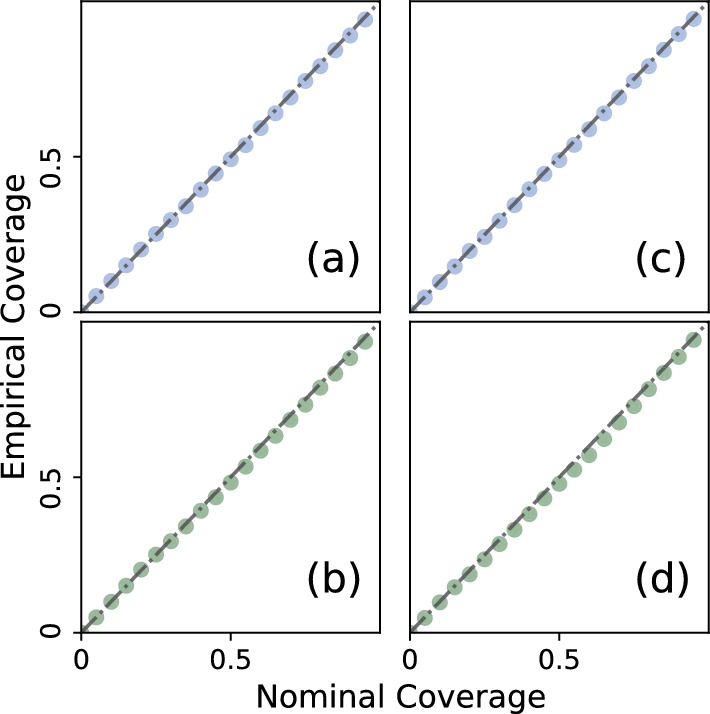
Empirical coverage of credible intervals plotted against nominal coverages of the MDN-ABC step for **a)** $\beta$ and **c)** $\gamma$ for the Erdős–Rényi network, and **b)** $\beta$ and **d)** $\gamma$ for the Log-normal network

While the HMC step in NA-MDN-ABC provides exact inferences, the MDN-ABC step is only an approximation. In order to validate the performance of the MDN-ABC step in the NA-MDN-ABC procedure, we will employ a coverage test similar to those proposed in Cook et al. (2006), Prangle et al. (2014) and Talts et al. (2018). For 5000 simulation instances, we drew $\theta _0$ from $p(\theta )$ and $(A_{t0},\phi _0)$ from $p(A_t,\phi |X)$. We then simulated a new dataset $Y_0$ based on these parameters and used these instances of $Y_0$ to draw 5000 posterior samples of $P(\theta ,A_t,\phi |X,Y_0)$ via NA-MDN-ABC. We then extracted the $\alpha \%$ credible intervals from these sampled posterior distributions, where $\alpha \in \{0,5,\ldots ,95\}$. As defined in Prangle et al. (2014), the “coverage property” is fulfilled for $\theta$ if the “true” values $\theta _0$ fall in the nominal $\alpha \%$ credible intervals $\alpha \%$ of the time (i.e. the empirical coverage of the nominally $\alpha \%$ credible interval is $\alpha \%$). In Fig. 4, we compare the nominal coverage to the empirical coverage for the contagion parameters $\beta$ and $\gamma$.

## Data application: Shark Bay Dolphins

In this section, we will apply our method to investigate the ongoing transmission of tattoo-skin disease (TSD) in a population of bottlenose dolphins in Shark Bay, Australia. This example is meant to be a demonstration of the NA-MDN-ABC method, rather than a fully accurate model of TSD transmission, so some simplifications are made for clarity.

Tattoo-skin disease is an infectious disease that primarily affects cetaceans. It is caused by a variety of cetacean poxviruses (Flom and Houk 1979; Bracht et al. 2006), and results in readily visible skin lesions in affected animals (Geraci et al. 1979). Previous studies of TSD have linked social behavior to the likelihood of being infected by TSD (Powell et al. 2020), and TSD prevalence appears to be much higher among nursing calves than weaned juveniles and adults. Although zoonotic transmission events have not been observed, humans commonly experience close contact with both wild and captive dolphin populations, making the transmission dynamics of TSD of potential public health interest. Furthermore, dolphins’ susceptibility to TSD may be exacerbated by factors such as water temperature and pollution (Van Bressem et al. 2003, 2009), making TSD prevalence a potential indicator of environmental stressors to marine habitats.

While previous studies have explored the epidemiological traits of TSD, we will apply MDN-ABC to study the individual-level transmission dynamics among the dolphins in Shark Bay, Australia. We will consider the rich dataset collected during the Shark Bay Dolphin Research Project, which tracked sightings of dolphins in Shark Bay starting from 1988, recording dolphin co-proximity events, disease status, and age. A public version of this dataset that contains the co-proximity events and disease statuses can be found at Powell et al. (2020). We made use of the data collected between June 1, 2010 and June 1, 2015, excluding observations on dolphins that were not seen in at least two sightings, at least 3 months apart. Additional data was kindly provided by the Mann Lab at Georgetown University, which denoted the age category of the dolphins at each sighting: calves (less than 3 years old), juveniles (3–10 years old), and adults. The age category of the calves, as well as the estimated birth date of individuals, was based off of sightings of the mothers, size, and ventral speckling (Krzyszczyk and Mann 2012; McEntee et al. 2023).

The Shark Bay dataset is notable in its capture of both a contact network of individuals and the individual-level propagation of an ongoing contagion. As with most observational data, it lacks exact event times (i.e. it is unknown exactly when dolphins transition between symptomatic and non-symptomatic states), and the reported co-proximity information serves as a proxy for the true infectious contact of interest (here, physical contact with the skin lesions of an affected dolphin). Thus, we must account for the uncertainty in the observations of the epidemic history as well as the contact network, making the dataset a good candidate for analysis using the NA-MDN-ABC. In the following sections, we will analyze the spread of TSD using a simple compartmental disease model and compare the results to known literature.

### Contagion model

While the exact biological means of transmission are not yet fully understood for TSD, we will model TSD as a discrete-time SIR process, with each time-step being 1 week.

Because it has been observed that TSD is more prevalent among calves and weaned juveniles than among adults (Powell et al. 2018), we consider three separate coefficients for transmissibility: $\beta _c$ for the per-contact transmission probability per time step for calves, $\beta _j$ for the per-contact transmission probability per time step for juveniles, and $\beta _a$ for the per-contact transmission probability per time step for adults. If at time *t* each node $i\in \mathcal {N}$ has a disease state $W_t^i\in \{S,I,R\}$ and age status $C_t^i \in \{\text{ calf, } \text{ juvenile, } \text{ adult }\}$, this can be expressed as:

6 $$\begin{aligned} \beta _c= & P(W^i_{t + \Delta t} = I \text{ via } j|W_t^i = S, W_t^j = I, C_t^i = \text{ calf}). \nonumber \\ \beta _j= & P(W^i_{t + \Delta t} = I \text{ via } j|W_t^i = S, W_t^j = I, C_t^i = \text{ juvenile}). \nonumber \\ \beta _a= & P(W^i_{t + \Delta t} = I \text{ via } j|W_t^i = S, W_t^j = I, C_t^i = \text{ adult}). \nonumber \\ \epsilon= & P(W^i_{t + \Delta t} = I \text{ spontaneous } | W_t^i = S). \end{aligned}$$

Here, $\{W_{t+\Delta t}^i = I \text{ via } j\}$ is defined as the event that node *i* is infected (caused to progress to exposed state) by infected neighbor *j*, while the event $\{W_{t+\Delta t}^i = I \text{ spontaneous }\}$ occurs when a node “spontaneously” acquires the contagion independently of any infectious contacts. This spontaneous probability of infection, defined as the “spark term” $\epsilon$, captures infectious sources that are not captured by the contact network (such as infection transmitted by a migratory individual who is never observed by researchers).

Note that some dolphins transition between age categories during the observation period. Although more accurate modeling of the age of dolphins may make use of photographic and social evidence from the dolphin sightings, we make some simplifying assumptions based on the available data. We considered weaned juveniles to be part of the “juvenile” category for the full duration of observation, unless that individual was later sighted as an adult. In those cases, we consider the juvenile as an adult starting from their first “adult” sighting. Calves are considered to become juveniles 3 years after their recorded birth-date. If the birth-date is not available, they become juveniles based on whichever of the following two events occurs first: (1) three years elapse after their first sighting as a first-year calf or (2) they are spotted as a weaned juvenile.

The duration of infectiousness is distributed as a Weibull distribution with shape $\gamma _a$ and scale $\gamma _b$. After this period of infectiousness, individuals transition to a permanent “recovered” state, at which time they are unable to infect others or be infected. Thus, if $U_i$ is the time that individual *i* would spend in the infectious period if individual *i* were to be infected, then:

7 $$\begin{aligned} U_i \sim \text{ Weibull }(\gamma _a, \gamma _b). \end{aligned}$$

The event times for the transition into each state is unknown for this population. Instead, the status of each dolphin is only given as a binary indicator of disease presence (visible skin lesions) upon each sighting. While false positives are unlikely, there may be false negatives in the sighting of disease (e.g., the diseased portion of the dolphin is not visible), but we will assume in this example that disease status is reliably reported. We also assume that the symptoms of disease coincide with the “infected” state in our model, such that infection can only occur upon contact with a symptomatic dolphin. Thus, if $Y_t^i$ is defined as the observed status of node *i* at time *t*:

8 $$\begin{aligned} Y_t^i = 1 \text{ if } W_t^i = I \text{, } Y_t^i = 0 \text{ otherwise }. \end{aligned}$$

To initiate our contagion, we treat all dolphins that were spotted with symptoms both before and after June 1, 2010 as the initial infected population.

For priors, we set $\beta _c \sim \text{ Uniform }(0,1)$, $\beta _j \sim \text{ Uniform }(0,0.01)$, $\beta _a \sim \text{ Uniform }(0,0.005)$, $\epsilon \sim \text{ Uniform }(0,0.001)$, $\gamma _a \sim \text{ Uniform }(0.2,5)$, and $\gamma _b \sim \text{ Uniform }(10,160)$. Note that the supports of the uniform priors for the transmissibility coefficients $\beta _j$ and $\beta _a$ are somewhat restrictive. In applications where there exist previous estimates in the literature for epidemic parameters, such estimates could be used to inform the priors. Here, the bounds of the priors were simply chosen such that the simulated epidemics tended to remain within realistic bounds. Due to the relatively large number of adults and juveniles in the population, this involved keeping the prior bounds for the per-contact transmissibility of adults and juveniles low, when compared to calves.

### Network reporting model

In the bottlenose dolphin population of Shark Bay, co-proximity is correlated closely with skin-contact, such that pairs of dolphins spotted together in co-sighting events are also much more likely to be observed engaging in direct contact such as rubbing and playing (Leu et al. 2020). However, co-sightings alone (the dataset that is publicly available) cannot be directly used to describe the transmission of TSD, as instances of co-proximity are not the actual potential transmission events for skin disease. Thus, we utilize the co-proximity events between dolphins to sample from the posterior distribution of the true underlying contact network.

Similarly to the example given in “Simulation study” section, we will assume that the underlying contact network is unweighted and undirected. Due to potential changes over time in the contact network of dolphins, we will infer a distinct contact network for each year of observation, for a total of 5 networks. We will consider the observed data to be the number of times each pair of dolphins was observed in a co-proximity event, such that $X_{ijw}$ is the number of times dolphins *i* and *j* were spotted in the same group during Year *w*. For each year, we model the number of counts as a Negative-Binomial distribution, with the parameters of the Negative-Binomial distribution differing across the 5 time-steps. Thus, our network model can be expressed as:

9 $$\begin{aligned} X_{ijw} | A_{ijw} = 0\sim & \text{ NegBin }(n_{0w}, p_{0w}, t_{0w}). \nonumber \\ X_{ijw} | A_{ijw} = 1\sim & \text{ NegBin }(n_{1w}, p_{1w}, t_{1w}). \nonumber \\ A_{ijw}\sim & \text{ Bernoulli }(\rho _w). \end{aligned}$$

While (Young et al. 2020) model a smaller set of dolphin data using a Poisson data model and we utilize a Poisson data model in the simulation study, in this data example, we expected that the Negative Binomial distribution could allow for greater flexibility in capturing the variability in the number of co-proximity events between dolphins.

We can then define the network observation parameters as $\phi = (n_{01},\ldots , n_{05}, p_{01},\ldots , p_{05}, n_{11},\ldots , n_{15}, p_{11},\ldots , p_{15}, \rho _1,\ldots , \rho _5)$. In Step 2 of Algorithm 1, we draw from the joint posterior of $P(A_{t1},\ldots , A_{t5}, \phi |X)$. This is accomplished using Hamiltonian Monte Carlo, implemented in STAN, following the method given by Young et al. (2020). In order to break the symmetry between edges and non-edges, and to improve the mixing properties of the algorithm, we will fix $p_{0w} = 1$ for all *w*. Our priors are defined as $n_{0w} \sim \text{ Gamma }(2,4)$, $n_{1w} \sim \text{ Gamma }(2,4)$, $p_{0w} \sim \text{ Gamma }(2,4)$, and $\rho _w \sim \text{ Beta }(1,20)$ for all *w*.

In order to validate the choice of model for the network reporting model, we employ the methodology set out in Young et al. (2020) and first proposed by Gelman et al. (1996). Following from Young et al. (2020), “discrepancy” is defined as:

10 $$\begin{aligned} D(X,A_{tw}^{'},\phi _w^{'}) = \sum _{ij} X_{ijw} \text{ log }\frac{X_{ijw}}{\langle \tilde{X}_{ijw}(A_{tw}^{'}, \phi _w^{'})\rangle }. \end{aligned}$$

**Fig. 5:**
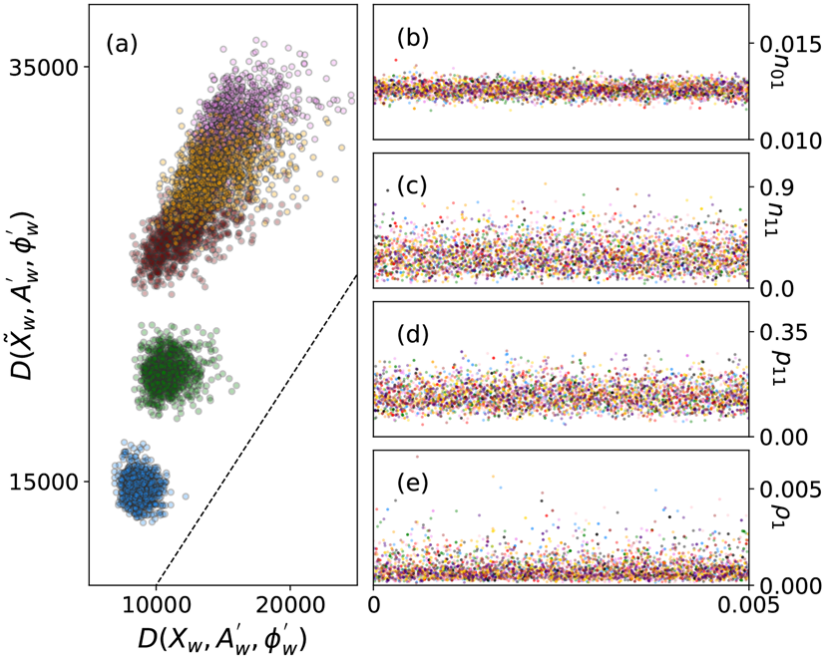
In Panel (**a**), discrepancy to the observed data is plotted against discrepancy to data generated from the model, with the dotted line denoting equality. In panels (**b**)–(**e**), samples from 10 independent HMC chains are shown for **b** $n_0$, **c** $n_1$, **d** $p_0$, and **e** $\rho$, for time $w = 0$

Here, $\langle \tilde{X}_{ijw}(A_{tw}^{'}, \theta _w^{'})\rangle$ is the mean of $X_{ijw}$ when the data is generated from the proposed model with the true underlying network set to $A_{tw}^{'}$ and the network reporting parameters set to $\phi _w^{'}$. Since a better model fit would imply a smaller discrepancy, we draw samples of $A_{tw}^{'}$ and $\phi ^{'}$ from the posterior distribution and calculate the discrepancy between $D(X_w,A_{tw}^{'},\phi ^{'})$ and $D(\tilde{X_w},A_{tw}^{'},\phi _w^{'})$, where $\tilde{X_w}$ is a network observation matrix simulated from the data-generating model. If the model is a good fit, we would expect that $D(X_w,A_{tw}^{'},\phi _w^{'})$ tends to be less than $D(\tilde{X_w},A_{tw}^{'},\phi _w^{'})$ (Young et al. 2020; Gelman et al. 1996). In Fig. 5, we plot the discrepancies for $w = 1,\ldots ,5$, varying colors between the discrepancies calculated for each aggregated duration of time.

Besides checking the goodness of fit for a model, it is also crucial to evaluate the quality of the Markov Chain samples. In Panels b) - e) of Fig. 5, we plot the samples from the HMC chain, for 10 chains, for the parameters $\phi ^{'}_0 = (n_{01}, n_{11}, p_{11}, \rho _1)$. The burn-in period was set to 1000 samples, after which 1000 samples were generated for the plots. Multiple modes or temporal trends may indicate poor mixing or insufficient burn-in, but such signs are absent. Similar results were observed for $w \in \{2,3,4,5\}$, and are shown in Fig. 10 in the “Appendix”. Note that only the network parameters $\phi$ are sampled during the initial HMC step; the epidemic parameters $\theta$ are sampled in the MDN-ABC step, and therefore do not have trace plots.

### MDN-ABC settings and results

Similarly to “Methods” section, we used a simple feed-forward neural network to learn our summary statistics. We set the dimension of the summary statistics to 20, and trained for 6 gamma-distributed components for our MDN. Once again, we generated 5 million samples for training, and 2.5 million samples for validation. We employed the same training strategy as “Simulation study” section.

**Fig. 6:**
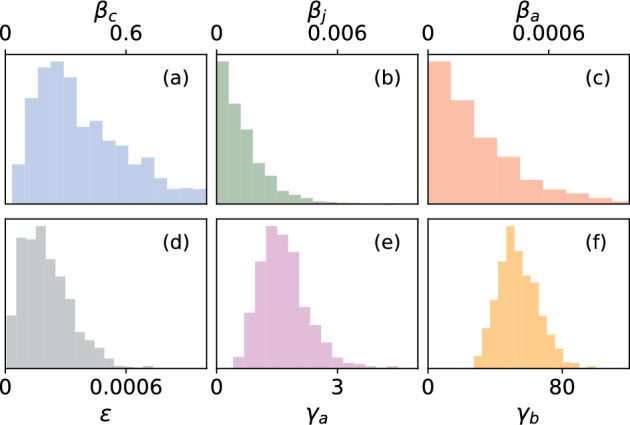
NA-MDN-ABC approximate posterior densities for contagion parameters, **a** $\beta _c$, **b** $\beta _j$, **c** $\beta _a$, **d** $\epsilon$, **e** $\gamma _a$, **f** $\gamma _b$

To draw the NA-MDN-ABC samples, we reused the training data and evaluated the summary statistics for each simulation instance. We then calculated the Euclidean distance between each set of summary statistics and the summary statistics yielded by the observed data, and selected the best $0.02\%$ of samples, resulting in approximately 1000 posterior samples. In Fig. 6, we show the NA-MDN-ABC posteriors for the contagion parameters. In particular, the posterior median for $\beta _c$ is nearly 10 times higher than the posterior median for $\beta _j$ and nearly 100 times higher than $\beta _a$. This indicates that calves are far more susceptible to TSD transmission than other demographic groups.

**Fig. 7:**
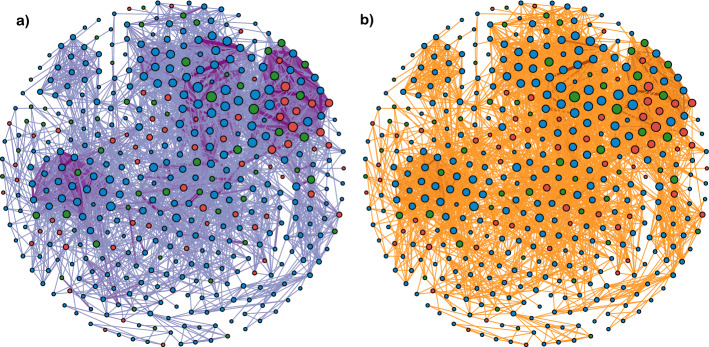
**a** Posterior distribution of contact network among dolphins, for time $w = 2$. Thicker edge weights and shades correspond to higher edge probabilities. Only edges with posterior probability greater than $10\%$ were included. The color of each node corresponds to the age category of the dolphin: adults in blue, juveniles in red, and calves in green. **b** Observed number of interactions between dolphins, with thicker edge weights and shades corresponding to higher counts of co-proximity events

Our inferences focus on obtaining inferences the parameters that govern epidemic spread. Thus, the contact network is treated as a nuisance parameter that is integrated over. However, because the posterior distribution we are approximating is the joint distribution $P(\theta , A, \phi |X,Y)$, we do obtain the posterior distribution of the contact network as a byproduct. In Fig. 7, we visualize the posterior distribution of the contact network of dolphins in year $w = 2$. Edge weight for each dyad (*i*, *j*) corresponds to the posterior probability of that edge’s existence: $P(A_{ij} = 1|\phi ,X)$. Node size for each node *i* corresponds to the sum of the posterior probabilities of all edges associated with a node: $\sum _j^N P(A_{ij}=1|\phi , X)$.

### Posterior predictive checks and comparison with literature

**Fig. 8:**
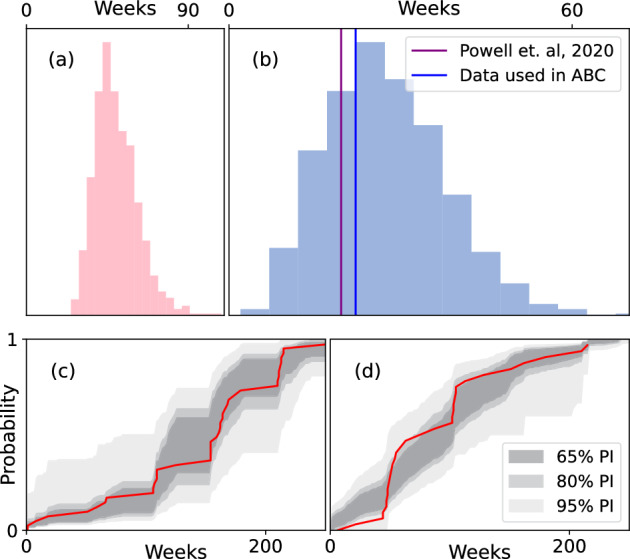
**a** NA-MDN-ABC approximate posterior density for infectious period. **b** Posterior predictive distribution of the mean difference between first and last infectious sightings, compared to Powell et al. (2018). **c** Predictive intervals for cumulative distribution function of initial infection times. **d** Predictive intervals for cumulative distribution function of interval between first and last infected sightings, for dolphins who were spotted with symptoms at least twice

**Fig. 10:**
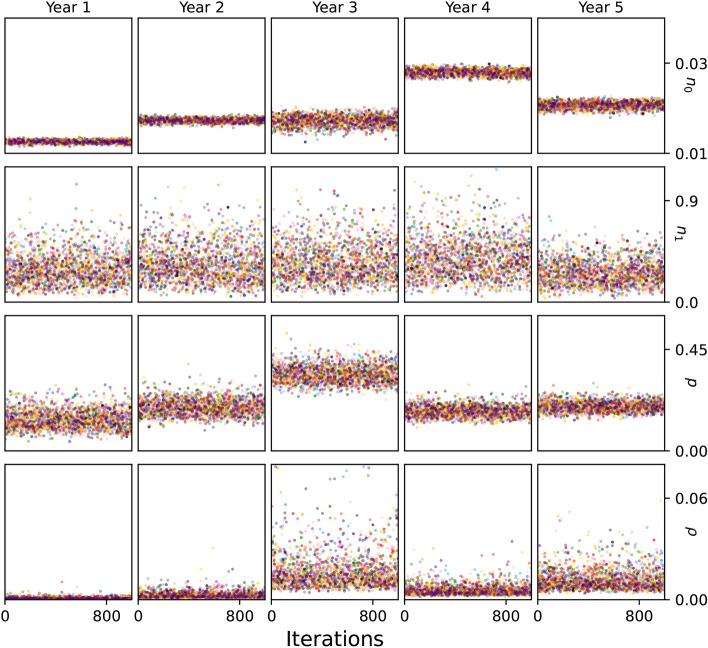
Trace plots for network parameters $\phi$, across all 4 years of observation

To our knowledge, no analysis has yet been conducted for the individual-level transmission dynamics of TSD among dolphins. Thus, there is no current standard of comparison for our inferences on $\beta _c$, $\beta _j$, and $\beta _a$. However, previous literature has already considered the infectious period of TSD. In bottlenose dolphins in the Sado Estuary, Portugal, the infectious period is estimated to be between 3 and 45.5 months (Van Bressem et al. 2003), although these long symptomatic periods may be due to various sources of pollution in the local environment. In Powell et al. (2018), which studied the Shark Bay bottlenose dolphins, it was estimated from photographic data that the infectious period lasts, on average, approximately 19.6 weeks. Using the posterior samples from our method, by taking the joint distribution of $(\gamma _a, \gamma _b)$ and calculating the posterior distribution of the mean infectious period from our NA-MDN-ABC posterior, we find that the posterior median for the mean infectious period is approximately 51.9 weeks, which appears initially to be a mismatch to the established literature. The posterior distribution of the mean infectious period is shown in the left panel of Fig. 8.

However, the infectious period in Powell et al. (2018) was determined by calculating the time difference between the first and last times the dolphins were spotted with symptoms of TSD. This is in contrast to our inferences, which aim to capture the full duration of infectiousness, regardless of when the infected dolphins were observed. Furthermore, while (Powell et al. 2018) focused on the same Shark Bay population we have analyzed, the authors employed a stricter set of inclusion criteria than we have used here (focusing analysis on a small pool of 10 well-observed individuals), as well as a photographic dataset not currently available publicly. In order to properly compare our model with these previous results, we will generate 1000 posterior predictive samples by sampling values of $\theta$ and $A_t$ from our NA-MDN-ABC posterior, and use these values to simulate new epidemic instances $Y_s$ from our model. From these simulations $Y_s$, we can calculate the posterior predictive distribution of the mean difference between the first and last times that infected dolphins are spotted with symptoms of TSD (the same method employed above). Furthermore, because the conclusions in Powell et al. (2018) stemmed from a different dataset from ours, we can perform an additional posterior predictive check to compare the fit of our model to the publicly available observed data used in our analysis. We will use the observed data used for our NA-MDN-ABC and once again calculate the mean difference between the first and last times that infected dolphins are spotted with TSD symptoms. This posterior predictive check is shown in Fig. 8, where the histogram denotes the posterior predictive distribution of this metric. Two vertical lines denote the mean “observed” symptomatic period derived from both (Powell et al. 2018) and the data we utilized.

Finally, we can observe the posterior predictive properties of two additional metrics. Due to missing event times, we cannot obtain the “true” epidemic curve for TSD in this population based purely on the observed data. However, we can calculate the distribution of two related metrics: (1) the first time each infected dolphin is initially spotted with symptoms, and (2) the time interval between the first and last times each infected dolphin is spotted with symptoms. Note that this second metric is closely related to the “mean observed symptomatic period” previously discussed; the difference is that we now consider the entire distribution, rather than the mean only. For both of these metrics, we can calculate the cumulative distribution function (CDF) on the observed data, as well as for each of our posterior predictive samples. In Fig. 8, we can obtain $\alpha \%$ predictive intervals for the CDFs derived from posterior predictive samples for $\alpha \in \{ 65,80,95 \}$, and evaluate how well these intervals describe the ground truth, plotted in red.

## Discussion

This paper considers the application of a network-augmented MDN-ABC for approximate Bayesian inference for epidemics spreading on noisily observed contact networks. In scenarios where the contact network is known or takes an analytically simple form, or where event times are known, it is possible to conduct exact likelihood-based estimation and inference, rendering ABC methods unnecessary. However, to the best of our knowledge, methods for Bayesian inference in the presence of both network uncertainty and event time missingness have not yet been proposed.

Due to the flexibility of the MDN-ABC and the network augmentation, our method provides a large degree of flexibility in modeling both the spread of contagion and the observation process on the network. Although inferences from MDN-ABC are approximate, MDN-ABC allows for posterior inference under complex models, such that researchers are not forced to choose between model expressiveness and analytical tractability.

There are various directions in which this work can be expanded. First, this paper is primarily concerned with settings where the frequency of disease testing is independent of the underlying contagion parameters. For example, in the Shark Bay Dolphin dataset, the researchers did not choose to specifically target diseased dolphins for observation. However, in some real-world settings, such as during the COVID-19 pandemic, individuals may seek out more frequent testing when they are symptomatic. In such cases, the dimensionality of the observed epidemic data *Y* would vary with values of parameters $\theta$, which would lead to the MDN having an input of varying dimensionality. Our current neural network architecture would be unable to handle this type of complexity, although this could be made possible with more specialized architectures, such as recurrent neural networks (Mesnil et al. 2013). Second, the separation of the network sampling step and the simulation of the epidemic proposed by our method requires the independence assumptions of “Methods” section. However, there may be situations where it is necessary to account for the simultaneous evolution of the network and the epidemic. In such cases, if observations on the network can be treated as informative of a “pre-epidemic” population, it may be possible to define and parameterize a model where the initial, pre-epidemic population is sampled, and the subsequent evolution of the network is accounted for in the epidemic simulation.

Our work primarily considers relatively simple network models. While we utilized the NA-MDN-ABC for a temporal network in “Data application: Shark Bay Dolphins” section, our model implicitly assumes that the network is memoryless; the previous relationship between two dolphins does not affect the probability of observing either an edge or a non-edge in future networks. More sophisticated models could incorporate previous network information to gain greater certainty about future networks. In addition, in “Data application: Shark Bay Dolphins” section, we model the observed data as a tensors of dyadic encounters, but the form of the reported data (group sightings of dolphins) could be a candidate for more modern hypergraph methods. Lastly, one might consider dynamic networks that better capture the the temporal changes in the social structures of individuals, rather than employing our strategy of aggregating all interactions over each year.

In the case of the Shark Bay Dolphin dataset, it may also be interesting to consider more advanced models for the spread of TSD. For example, we modeled TSD as a simple SIR disease, which assumes that the incubation period for TSD is short relative to our simulation time-steps (1 week). However, it may be more appropriate to model TSD as an SEIR disease, with an asymptomatic, yet infectious, exposed “E” state as well. Furthermore, our model does not consider the possibility of different strains of TSD with varying infectivity spreading in the population, though this can be easily implemented.

Lastly, we utilized a simple rejection ABC algorithm for the sake of demonstration. More specialized applications of NA-MDN-ABC may choose to employ more sophisticated algorithms, including Monte Carlo Markov Chain ABC (MCMC-ABC) and Sequential Monte Carlo (SMC-ABC) (Marjoram et al. 2003; Sisson et al. 2007). The primary improvement these algorithms offer is the ability to use previously accepted proposals to inform future proposals, thus requiring less simulations. Because MDN-ABC requires a large set of training simulations to train the MDN, we consider it a natural extension to simply re-use these training simulations for rejection ABC. However, once the MDN is trained and the summary statistics are obtained, implementation of other ABC algorithms is straightforward.

Every epidemic is unique. The temporal and spatial granularity at which cases are observed, the degree of knowledge about individual contact patterns, and the coverage of testing are all dependent on political and social dimensions of the affected population and biological characteristics of the contagion. In the study of epidemics on networks, there remains a need for methods that are able to bridge the gap between theory and obtainable data. To this purpose, we have proposed NA-MDN-ABC as a flexible tool for Bayesian inferences. By delegating the definition of summary statistics to a neural network, this method can be readily extended to a variety of data observation settings to study and control real-world epidemics.

## Figures and Tables

**Fig. 9:**
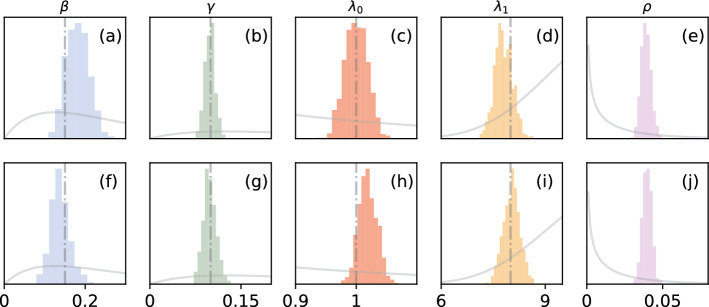
Posterior samples generated from NA-MDN-ABC, utilizing SMC-ABC instead of rejection ABC. Panels **b**–**e** display the results for the Erdős–Rényi network, and panels **f**–**j** show results for the log-normal distributed network. “True” values are marked with a vertical line. Prior densities are shown in gray (prior densities for $\lambda _0$ and $\lambda _1$ have been multiplied by an additional factor of 10 for visibility)

## Data Availability

The Shark Bay Dolphin Dataset analyzed in this paper is publicly available on Dryad Powell et al. (2020). Additional supplementary information regarding the ages of individual dolphins was kindly provided by the Mann Lab at Georgetown University (https://www.monkeymiadolphins.org/). The code utilized in this paper is available on GitHub (https://github.com/onnela-lab/abc-uncertain-networks).

## Appendix

### Justification of sampling scheme

We consider the sampling of the joint posterior $P(\theta , A_t, \phi |X,Y)$. If it can be assumed that (1) $X \perp Y |A$, (2) $\theta \perp \phi$, and (3) $\theta \perp A$, the following holds true:

11 $$\begin{aligned} \begin{aligned} P(\theta ,A,\phi |X,Y)&\propto P(X,Y|\theta ,A_t,\phi )P(\theta ,A_t,\phi ) \\&= P(X|A,\phi )P(Y|\theta ,A,\phi )P(A,\phi )P(\theta )\\&= P(Y|\theta , A, \phi )P(X, A, \phi )P(\theta )\\&\propto P(Y|\theta , A, \phi )P(A,\phi |X)P(\theta ). \end{aligned} \end{aligned}$$

In Algorithm 1, an instance $\phi '$ and $\theta '$ are sampled from their respective priors $P(\phi ')$ and $P(\theta ')$.

First, $A'$ must be drawn from the posterior $P(A,\phi |X)$. Following the work and notation put forth in Young et al. (2020), when dyadic independence can be assumed, this posterior can be expressed as:

12 $$\begin{aligned} \begin{aligned} P(A,\phi |X)&\propto P(X|\phi ,A) P(A|\phi )P(\phi )\\&= P(\phi )\prod _{(i,j)} P(X_{ij}|A_{ij},\phi )P(A_{ij}|\phi ) \end{aligned} \end{aligned}$$

For many simple models, this likelihood is computationally tractable. For example, for the simulation study investigated in “Simulation study” section, the distribution $P(X_{ij}|A_{ij},\phi )$ takes the form of a Poisson likelihood and $P(A_{ij}|\phi )$ is a Bernoulli distribution. Thus, samples of $(A',\phi ')$ can be drawn using a variety of methods, including MCMC and HMC.

Once $A'$ is sampled, $Y'$ can be simulated from an epidemic model with the unknown likelihood $P(Y'|\theta ',A',\phi ')$.

Let $(\theta ',Y',A',\phi ')$ be a single instance of the parameters sampled from Algorithm 1. We define *d* as a distance function and $\delta$ as a tolerance level, and accept this proposed sample of the parameters if the distance function between the observed data *Y* and the sampled data $Y'$ is below the tolerance level. We define $S(Y')$ as the vector of summary statistics computed from $Y'$; in this manuscript $S(Y')$ is the output of the MDN. We can now set $D_{\delta ,Y}(Y') = 1$ if $||d(S(Y') - S(Y))||<=\delta$ and 0 otherwise.

We can then express the joint distribution of the accepted values of $(\theta ',Y',A',\phi ')$ as:

$$\begin{aligned} \pi (\theta ',Y',A',\phi ') = P(Y'|\theta ',A')P(\theta ')P(A',\phi '|X)D_{\delta ,Y}(Y'). \end{aligned}$$

Integrating over $Y'$, we obtain:

$$\begin{aligned} \pi (\theta ',A',\phi ') = \int P(Y'|\theta ',A')P(\theta ')P(A',\phi '|X)D_{\delta ,Y}(Y') dY'. \end{aligned}$$

This distribution forms the sampled ABC posterior. Note that in the limiting case where $S(Y) = Y$ and $\delta = 0$, samples are accepted only when $Y' = Y$. Thus, we set $D_{\delta ,Y}(Y') = 1$ only if $Y' = Y$. In this limiting case, the ABC posterior simplifies to the true posterior:

$$\begin{aligned} \pi (\theta ',A',\phi ') = P(Y|\theta ',A')P(A',\phi '|X)P(\theta ')\propto P(\theta ',A',\phi '|X,Y). \end{aligned}$$

### Simulation results, utilizing SMC-ABC

In “Methods” section, we implemented rejection ABC for posterior sampling. However, more advanced ABC algorithms are able to use previous simulations to inform subsequent proposals. Here, we sample from the posterior by utilizing SMC-ABC (Toni et al. 2009) instead of rejection ABC; for a direct comparison, we allowed the SMC-ABC to run until the highest discrepancy in the sample equaled the acceptance threshold used in “Methods” section. These samples are visualized in Fig. 9.
